# Supplementary material for: Maternally derived anti-helminth antibodies predict offspring survival in a wild mammal
Source: Proc Biol Sci. 2020 Nov 25;287(1939):20201931. doi: 10.1098/rspb.2020.1931 (PMC7739501; doi:10.1098/rspb.2020.1931)
Supplement: Supplementary information [file rspb20201931supp1.pdf]

Supplementary Material for:

Maternally derived anti-helminth antibodies predict offspring survival in a wild mammal

Alexandra M. Sparks, Adam D. Hayward, Kathryn Watt, Jill G. Pilkington, Josephine M. Pemberton, Susan E. Johnston, Tom N. McNeilly, Daniel H. Nussey

Proceedings of the Royal Society B

DOI: 10.1098/rspb.2020.1931

http://dx.doi.org/10.1098/rspb.2020.1931

Supplementary Methods

These ELISA methods were developed from methods detailed in [1,2] with the addition of the total IgG assay. We used *T. circumcincta* L3 somatic antigen, provided by the Moredun Research Institute, as the capture antigen for the *T. circumcincta* assays diluted to 2µg/ml in 0.06M Carbonate buffer at pH 9.6. For the total IgG assay, we diluted rabbit anti-sheep IgG (Bio-Rad 5184-2104) to 2µg/ml in 0.06M Carbonate buffer at pH 9.6. 50µl of the diluted capture antibody/antigen was added to each well of a Nunc-immuno 96-microwell plate, which was covered and incubated at 4ºC overnight. After washing the wells three times in Tris-buffered saline-Tween (TBST) using a plate washer, 50µl of the Soay sheep plasma sample diluted to 1:50 for anti- *T. circumcincta* IgA and IgE, 1:12800 for anti- *T. circumcincta* IgG and 1:819200 for total IgG was added to each well. The plates were then covered and incubated at 37ºC for 1 hour. Plates were then washed five times with TBST and 50µl per well of rabbit anti-sheep IgA detection antibody conjugated to horseradish peroxidase (HRP) (AbD Serotec AHP949P) diluted 1:16000 was added to the anti-*T. circumcincta* IgA assay and 50µl per well of rabbit anti-sheep IgG detection antibody conjugated to HRP (AbD Serotec 5184-2504) diluted 1:16000 was added to the anti-*T. circumcincta* IgG and total IgG assays. For the anti-*T. circumcincta* IgE assay, 50µl per well of anti-sheep IgE (mouse monoclonal IgG1, clone 2F1, provided by the Moredun Research Institute) diluted 1:100 was added, followed by 1 hour incubation at 37ºC, five washes with TBST and then 50µl per well of goat anti-mouse IgG1-HRP detection antibody (AbD Serotec STAR132P) diluted to 1:8000 in TBST was added. All plates were then incubated at 37ºC for 1 hour. Plates were then washed five times with TBST and 100µl of SureBlue TMB 1-Component microwell peroxidase substrate (KPL) was added per well and left to incubate for 5 minutes in the dark at 37ºC. Reactions were stopped by adding 100µl per well of 1M hydrochloric acid and optical densities (OD) were read immediately at 450nm using a Thermo Scientific GO Spectrophotometer.

All results were recorded as OD values. In order to minimise confounding birth year effects with plate to plate variation, each plate included samples from two years paired at random. All plates were run in duplicate and duplicate sample ODs were removed if the coefficient of variation was > 0.2 and the difference between ODs was greater than 0.2. We also checked the correlation of ODs across duplicate plates and re-ran both plates if $r < 0.8$. To reduce error due to within-plate variation, per plate we included two sample free wells (50µl TBST) as blanks and two wells of positive controls. Positive controls for the IgE assay were serum from ewes trickle infected with *T. circumcincta* and for the IgA and IgG assay were plasma from normal healthy non-immunised domestic sheep. For subsequent analyses, the mean optical density ratio of each sample was taken according to this formula:

$$OD = \frac{(\text{sample OD} - \text{blank OD})}{(\text{positive control OD} - \text{blank OD})}$$

Where the numerator was set to zero if the blank OD was greater than the sample OD in order to avoid negative values. The number of samples that failed quality control per assay was 16 for anti-*T. circumcincta* IgA, 11 for anti-*T. circumcincta* IgE, 17 for anti- *T. circumcincta* IgG, and 14 for total IgG.

Our neonatal antibody dataset initially comprised of 3,491 lambs which had blood samples taken in the lambing seasons between 1990 and 2015. No April samples or data were collected in 2001 due to foot-and-mouth disease precautions. Five individuals had two samples, and these duplicates were removed, taking the first measurement if samples were collected on different days, or picking one of the duplicates at random if not. One lamb born late in the season (July) was removed from the dataset. We further restricted analyses to neonates caught within 10 days of birth for three reasons: first, to accurately estimate birth weight from capture weight since the relationship between age and capture weight changes after this; second, due to a sharp decline in antibody levels in older lambs (Figure S1); and third, to account for low sample sizes in older capture age groups (n = 85). Our final dataset comprised of neonatal antibody measures for 3,379 lambs from 845 mothers, 52% of the lambs were female and 24% were twins.

All samples were assayed between 13/5/2016-26/7/2016 but were collected over many years and stored at -20°C until they were assayed. We checked for possible signatures of sample degradation over time since collection but we found no positive temporal trend in levels of any of the four antibody measures as expected with sample degradation. In fact we found evidence for negative, but weak, temporal trends in the levels of the four antibody measures with sample year (IgA: β=-0.005 ± 0.001 SE, F_1= 13.540, p<0.001; IgE: β=-0.015 ± 0.002 SE, F_1= 68.811, p<0.001; IgG: β=-0.002 ± 0.001 SE, F_1= 9.182, p=0.002; total IgG: β=-0.001 ± 0.001 SE, F_1= 3.915, p=0.048), however, there was considerable variation between years (Figure S2) suggesting sample degradation was not present.

Mammals have five classes (isotypes) of antibodies: IgA, IgE, IgD, IgG and IgM. IgG is the most abundant isotype in serum, and is effective at neutralising toxins, activating complement and the agglutination and opsonisation of pathogens. IgM is the second-commonest isotype in serum, and is the major isotype produced following first exposure to an antigen. IgM forms pentamers making them effective activators of the complement system but prevents them readily leaving the bloodstream. The

third most common isotype in serum is IgA, which is the most common isotype in secretions including mucosal surfaces and it acts as a strong neutralising antibody and can agglutinate pathogens. IgD can be found on B cells and this isotype tends not to be secreted. Finally, IgE is present at very low concentrations in serum, has the lowest half-life of all antibodies, but is key in mediating acute inflammatory responses. Colostrum is rich in immunoglobulins, predominantly IgG in ewes, followed by IgM and IgA. In milk, the concentration of immunoglobulins drops and IgG continues to predominate but IgA becomes the second most dominant isotype [3]. In peri-parturient ewes in this population we found uniformly positive correlations between anti-Tc IgG, IgA, IgM and total IgG, IgA and IgM, suggesting that maternally-transferred antibody levels are likely to be positively correlated in neonates [4].

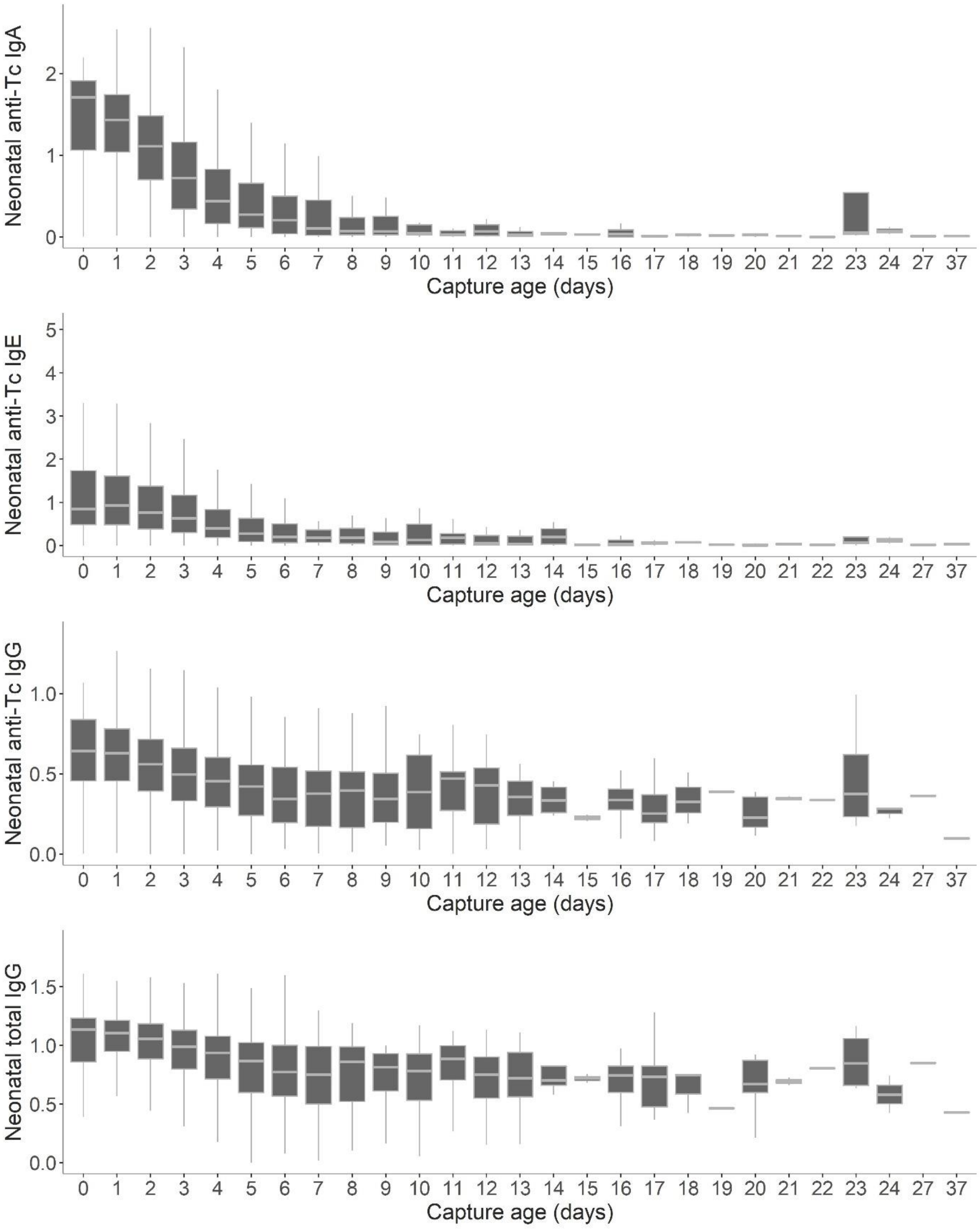

Figure S1. Boxplots of raw data showing associations between antibody levels and capture age in days since birth for anti-*Teladorsagia circumcincta* IgA, IgE, IgG and total IgG in neonatal wild Soay sheep. Boxes show the median and the interquartile range (IQR) with whiskers extending from the hinges to values no further than 1.5*IQR from the hinge (outliers not shown).

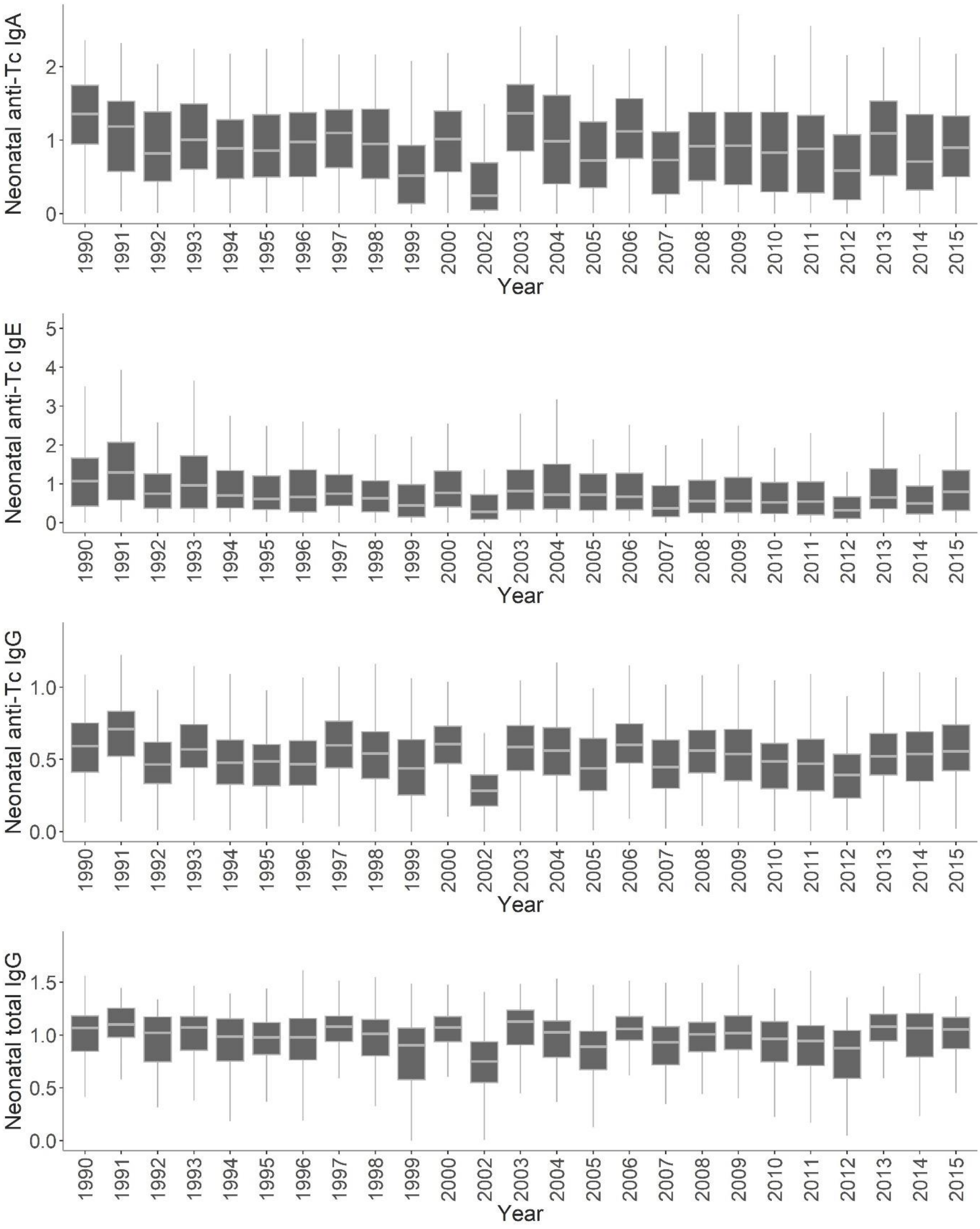

Figure S2. Boxplots of raw data showing associations between sample year and antibody levels (anti-*Teladorsagia circumcincta* IgA, IgE, IgG and total IgG) in neonatal wild Soay sheep to investigate presence of sample degradation over time. Boxes show the median and the interquartile range (IQR) with whiskers extending from the hinges to values no further than 1.5*IQR from the hinge (outliers not shown). Regression analyses revealed a weak negative association between antibody levels and sample year (see Supplementary Methods) which does not support evidence for sample degradation over time.

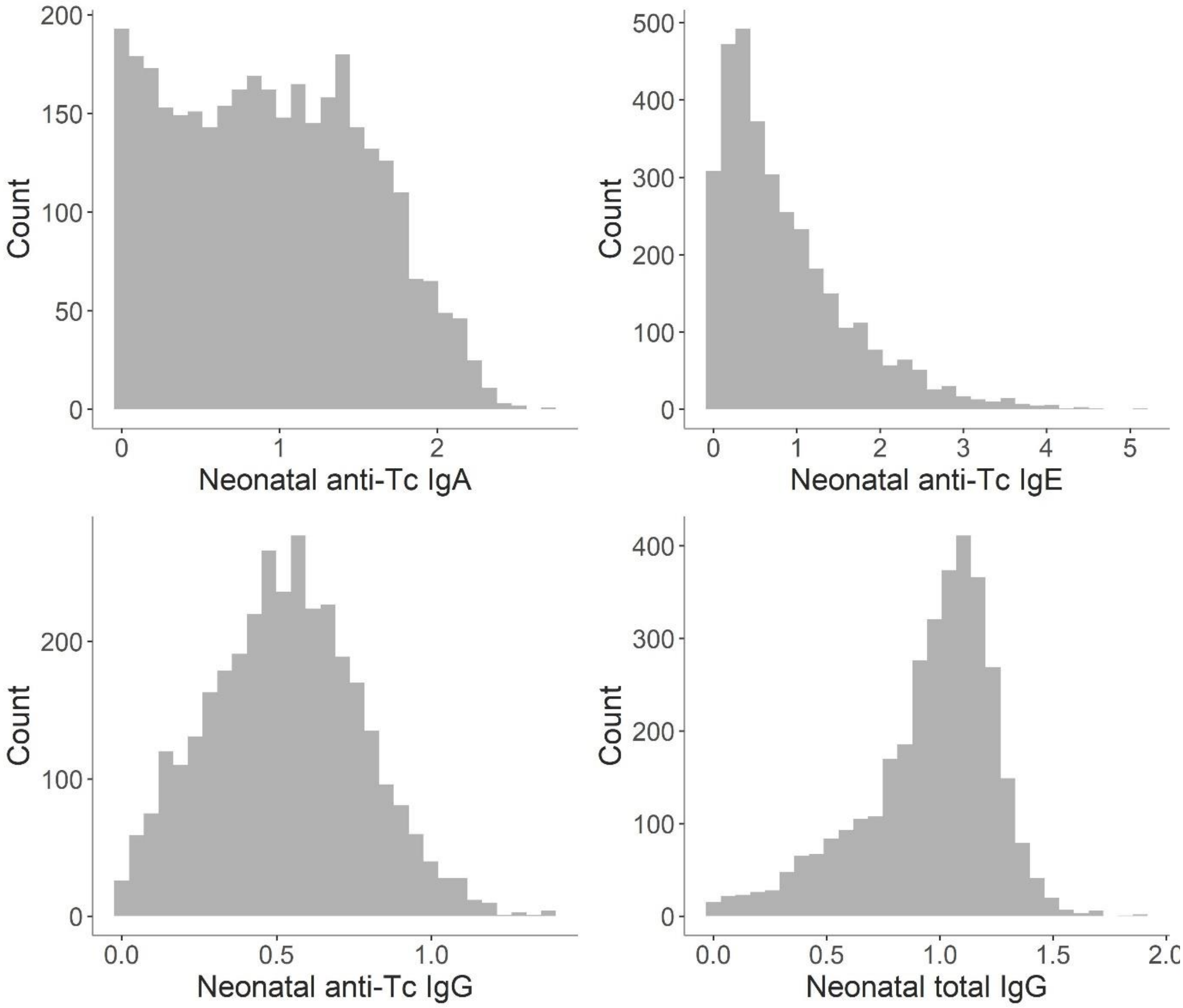

Figure S3. Histograms of anti-*Teladorsagia circumcincta* IgA, IgE, IgG and total IgG levels in neonatal Soay lambs.

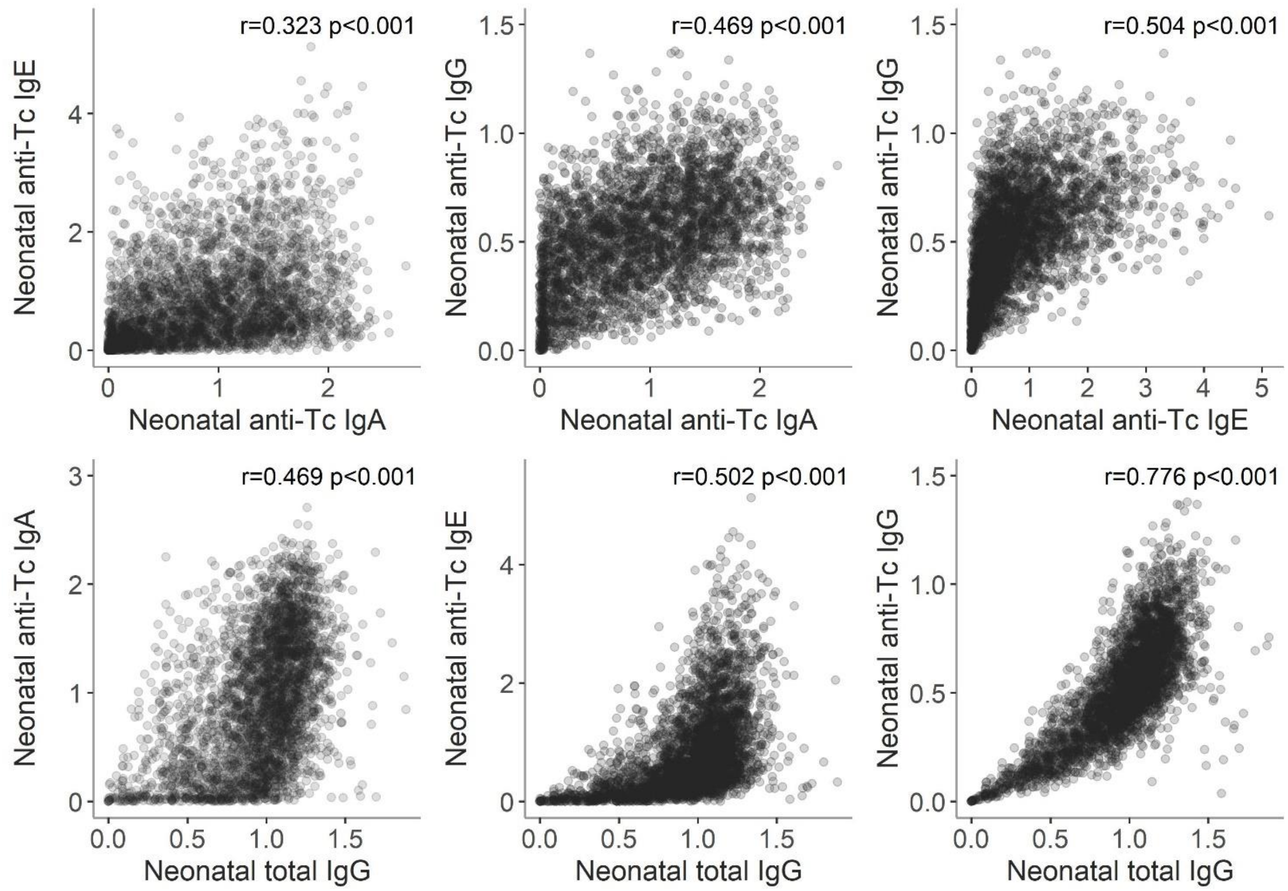

Figure S4. Scatterplots of raw data showing correlations between anti-*T. circumcincta* IgA, IgE, IgG and total IgG antibody levels in neonatal Soay sheep lambs. Correlation coefficients (r) and p-values quoted are from Pearson correlation tests.

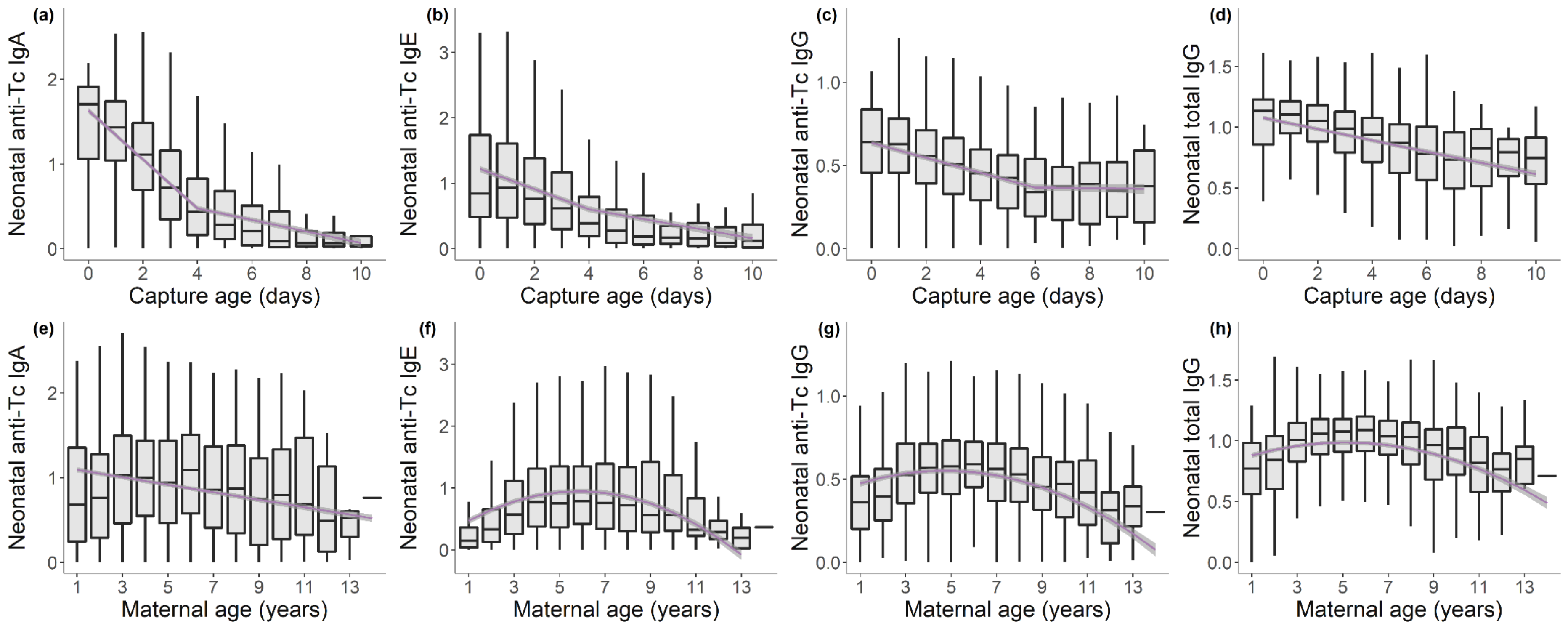

Figure S5. Associations between capture age (a-d) and maternal age (e-h) and neonatal anti-*T. circumcincta* IgA, IgE and IgG and total IgG levels. Plots show raw data with LMM predictions and standard errors estimated for female singleton lambs with average values for all continuous fixed effects in the minimal model (Table S3). Boxes show the median and the interquartile range (IQR) with whiskers extending from the hinges to values no further than 1.5*IQR from the hinge (outliers not shown).

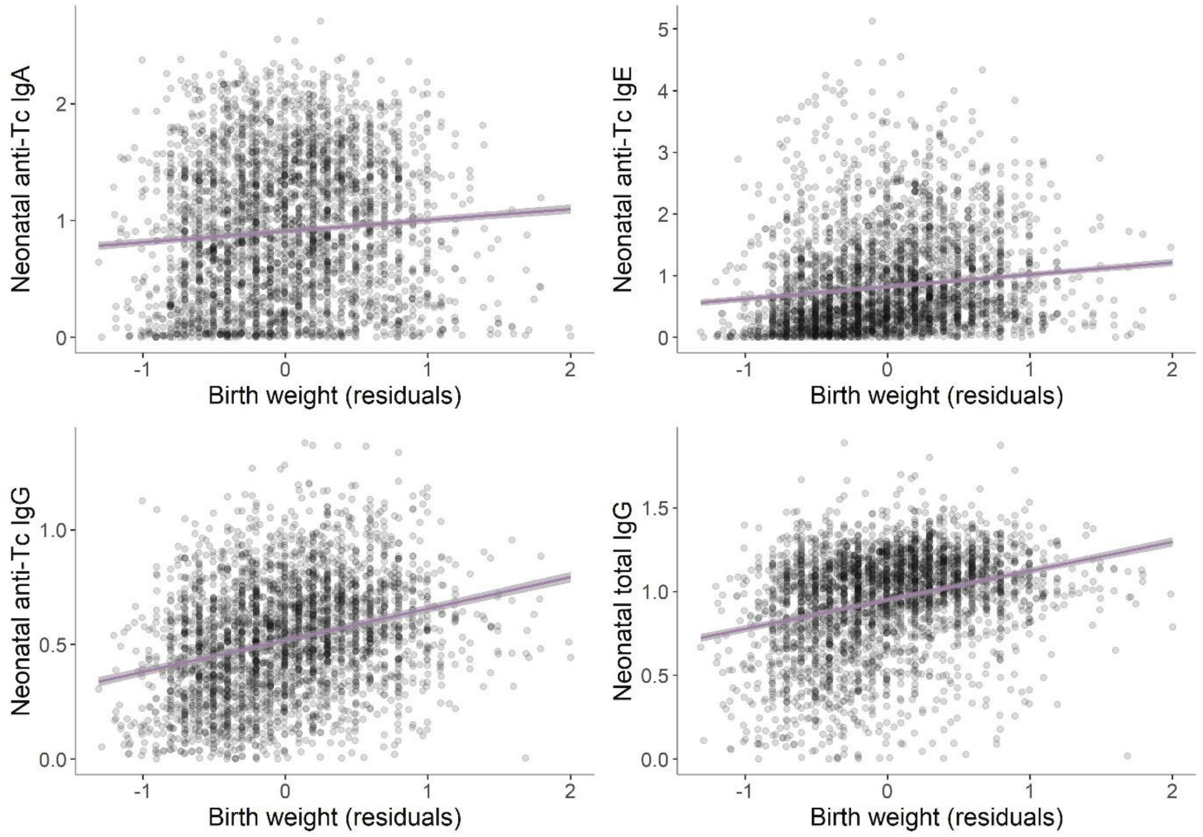

Figure S6. Associations between birth weight (corrected for capture age, see Methods) and neonatal anti-*T. circumcincta* IgA, IgE and IgG and total IgG levels. Plots show raw data with LMM predictions and standard errors estimated for female singleton lambs with average values for all continuous fixed effects in the minimal model (Table S3).

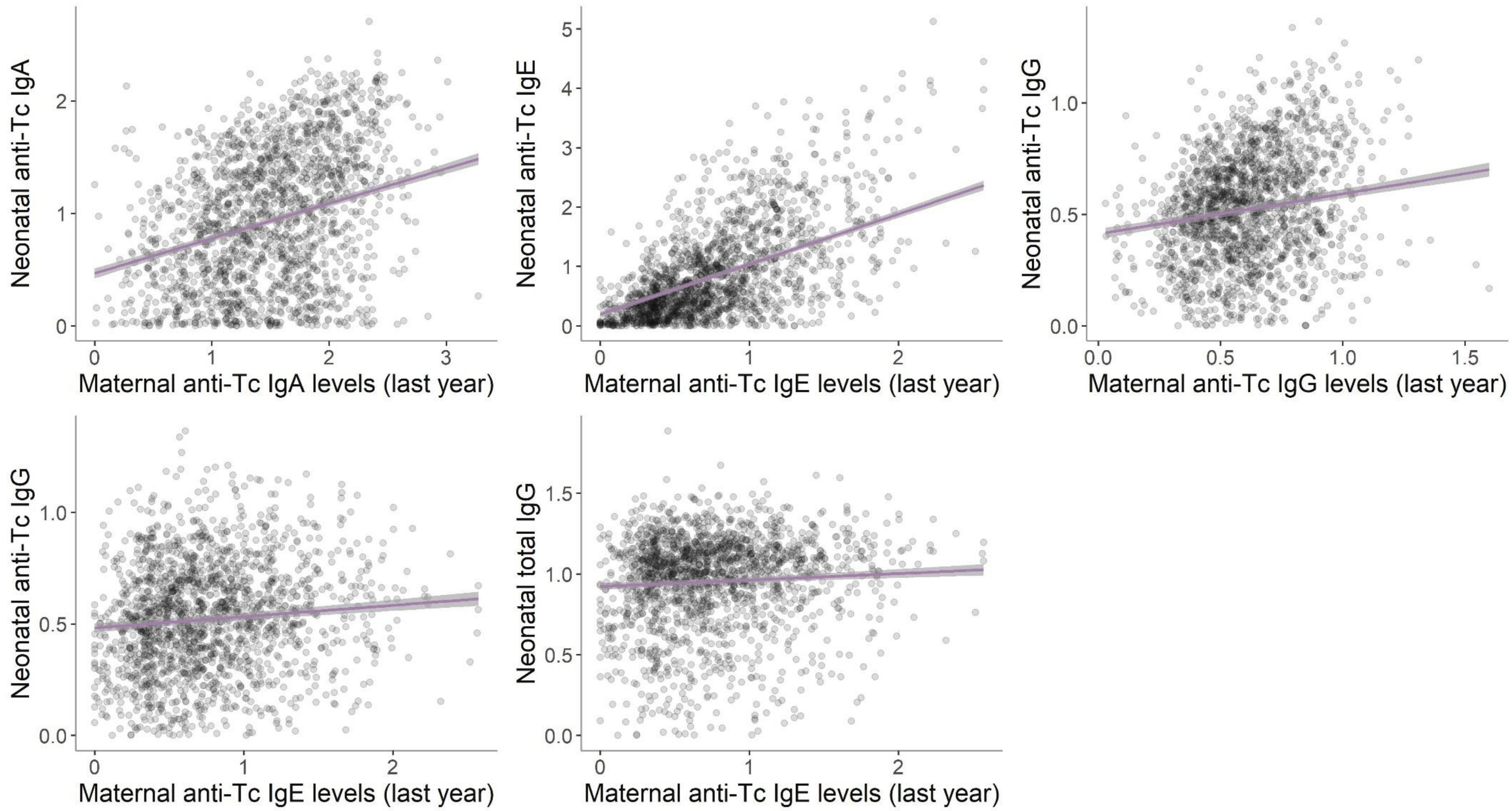

Figure S7. Associations between maternal anti-*T. circumcincta* plasma antibody levels measured in the previous year and her offspring's neonatal anti-*T. circumcincta* IgA, IgE and IgG and total IgG levels. Plots show raw data with LMM predictions and standard errors estimated for female singleton lambs with average values for all continuous fixed effects in the model (Table S3).

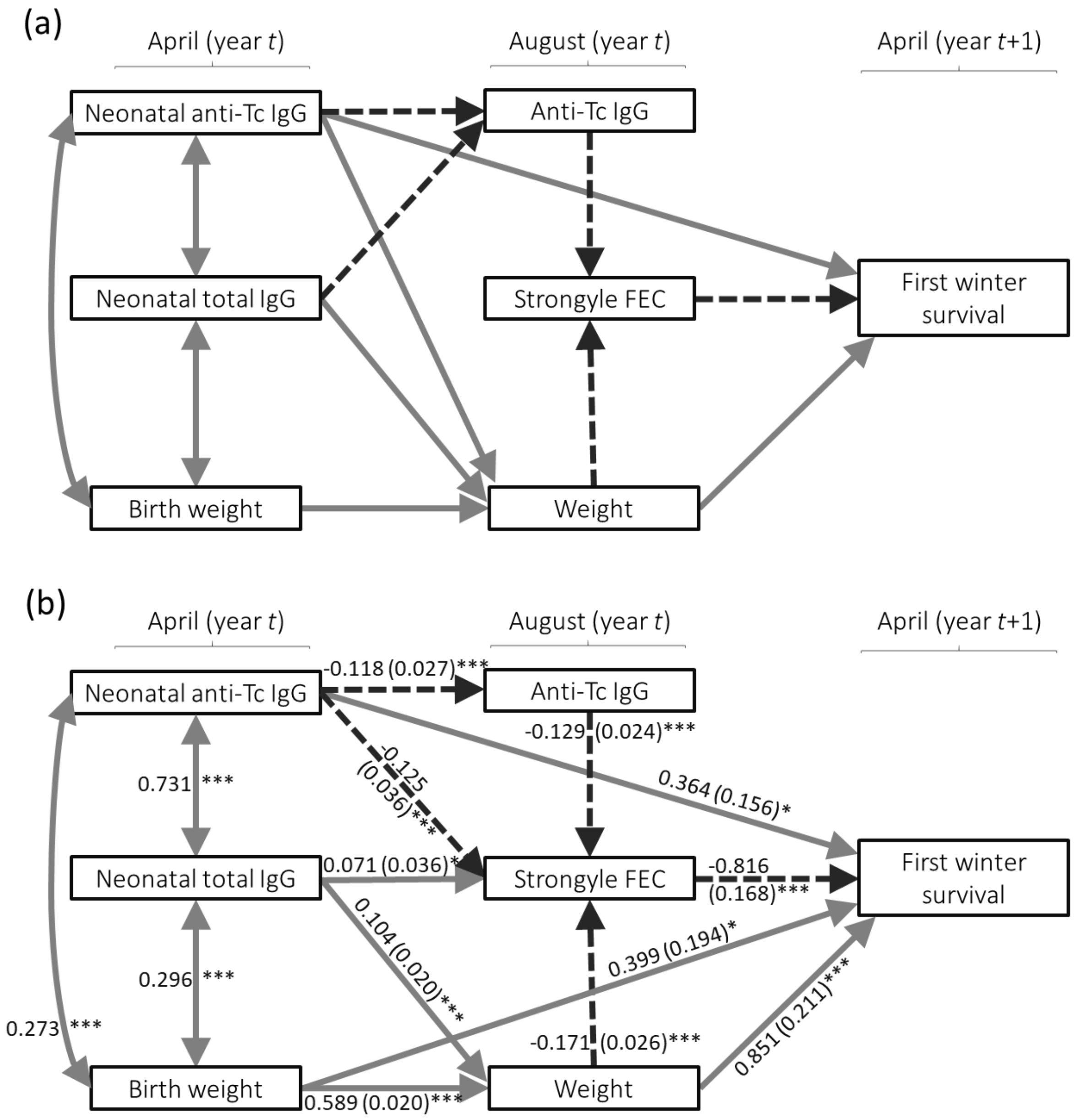

Figure S8. (a): *A priori* structural equation model linking neonatal anti-*T. circumcincta* and total IgG antibodies to first winter survival. (b): Final structural equation model with values on arrows indicating standardised path coefficients with standard errors in brackets, except for the bidirectional links between neonatal measures which show correlation coefficients. This is an alternative model where anti-*T. circumcincta* IgG is included as the August antibody over anti-*T. circumcincta* IgA. Missing paths in the *a priori* model were added where indicated using Shipley's test of d-separation and unsupported paths were removed based on p-values ≥0.05. Effects are separated into positive (grey full lines) and negative (black dashed lines) effects with p-values indicated by * (*** = P<0.001, **=P<0.01 and * P<0.05). This model was based on 1,391 lambs which had data on neonatal antibody levels, August measures and first winter survival.

Table S1. Fixed and random effects fitted in the models investigating associations between neonatal antibody levels and August weight, August strongyle FEC, August anti-*T. circumcincta* antibody levels and neonatal and first winter survival in Soay sheep.

Response variable	Error distribution	Sample size	Fixed effects	Random effects
August weight	Gaussian LMM (lme4)	1770	Sex, twin status, birth weight, age (days), maternal age (linear, quadratic)	Year, maternal identity
August strongyle FEC	GLMM with "nbinom2" negative binomial distribution with zero inflation (glmmTMB)	1528	Sex, twin status, age (days), August weight, August anti-Tc IgA	Year, maternal identity
August anti-Tc IgA	Gaussian LMM (lme4)	1773	Sex, twin status, birth weight, age (days)	Year, maternal identity
August anti-Tc IgE	Gaussian LMM (lme4)	1771	Sex, twin status, birth weight, age (days)	Year, maternal identity
August anti-Tc IgG	Gaussian LMM (lme4)	1775	Sex, twin status, birth weight, age (days)	Year, maternal identity
Neonatal survival	GLMM binomial (lme4)	2910	Sex, twin status, birth weight, maternal age	Maternal identity
First winter survival	GLMM binomial (lme4)	1630	Sex, August weight, Sex*August weight	Year, maternal identity

Table S2. Paths included in the structural equation model analysis and the evidence for each association.

Path	Direction	References
Neonatal anti-Tc IgG → First winter survival	Positive	This study
Neonatal anti-Tc IgG → August anti-Tc IgA	Negative	This study
Neonatal anti-Tc IgG → August weight	Positive	This study
Neonatal total IgG → August anti-Tc IgA	Negative	This study
Neonatal total IgG → August weight	Positive	This study
Birth weight → August weight	Positive	[5]
August anti-Tc IgA → August strongyle FEC	Negative	[1]
August strongyle FEC → First winter survival	Negative	[6,7]
August weight → August strongyle FEC	Negative	[8,9]
August weight → First winter survival	Positive	[10]

Table S3. LMM results of the final minimal model for neonatal anti-*T. circumcincta* IgA, IgE and IgG levels and neonatal total IgG levels in St Kilda Soay sheep. Included are the estimated effects (est), standard error (SE) and the significance of fixed effects based on a likelihood ratio test (LRT, p-value) where d.f.=1. Random effects are quoted from the minimal model excluding added fixed effects. Dropped fixed effects show the significance of adding dropped terms back to the minimal model. For the added fixed effects, each of the mother's antibody measures (of the same isotype) taken in the August preceding was added separately to the minimal model (as not all mothers had antibody levels measured in the previous summer). Where the quadratic maternal age terms are significant, significance tests are for both the linear and quadratic terms in together. t indicates a threshold model that best fits the capture age relationship, in IgA and IgE models this is at day 4 and in the IgG model this is at day 6. The raw mean of neonatal anti-Tc IgA, IgE, IgG and total IgG was 0.949, 0.870, 0.527 and 0.953 and the variance of neonatal anti-Tc IgA, IgE, IgG was 0.368, 0.619, 0.061 and 0.087.

	**Neonatal anti-Tc IgA**				**Neonatal anti-Tc IgE**				**Neonatal anti-Tc IgG**				**Neonatal total IgG**			
	n=3213				n=3218				n=3212				n=3214			
variables	est	SE	LRT	P	est	SE	LRT	P	est	SE	LRT	P	est	SE	LRT	P
fixed effects																
intercept	1.862	0.037			0.649	0.069			0.546	0.026			0.947	0.031		
sex (male)									-0.024	0.006	13.444	<0.001	-0.029	0.009	10.380	0.001
twin	0.127	0.023	29.934	<0.001	0.073	0.031	5.579	0.018	0.044	0.012	13.162	<0.001	0.045	0.015	8.569	0.003
capture age (days)													-0.046	0.003	244.820	<0.001
capture age: ≤ t days	-0.289	0.008	1032.100	<0.001	-0.154	0.010	223.740	<0.001	-0.045	0.003	225.290	<0.001				
capture age: > t days	-0.068	0.008	63.673	<0.001	-0.074	0.010	51.727	<0.001	-0.002	0.008	0.056	0.813				
birth weight	0.095	0.017	30.007	<0.001	0.196	0.024	67.023	<0.001	0.138	0.009	208.520	<0.001	0.173	0.012	197.130	<0.001
maternal age	-0.044	0.004	152.280	<0.001	0.237	0.018	-	-	0.052	0.007	-	-	0.065	0.009	-	-
maternal age^2					-0.020	0.001	186.260	<0.001	-0.006	0.001	92.832	<0.001	-0.006	0.001	70.260	<0.001
dropped fixed effects																
sex			0.686	0.408			0.668	0.414								
maternal age^2			1.506	0.220												
added fixed effects																
maternal anti-Tc Ig	0.311	0.024	142.540	<0.001	0.843	0.037	361.150	<0.001	0.180	0.026	39.151	<0.001			2.128	0.145
random effects																
maternal ID	0.121				0.304				0.021				0.010			
year	0.009				0.026				0.004				0.004			
plate	0.003				0.007				0.002				0.003			
residual	0.116				0.161				0.027				0.055			

Table S4. AIC comparisons of models investigating different capture age relationships with antibody levels in neonatal Soay sheep. Different capture age relationships include linear and quadratic terms, plus threshold age models with a single threshold at day 1 to 9 ("t=") for anti-*T. circumcincta* IgA, IgE, IgE and total IgG levels. Models included birth weight, maternal age (quadratic), sex and twin status of the lamb as fixed effects in addition to maternal identity, year and plate as random effects. The best fitting models with the lowest AIC for each isotype are highlighted in bold.

Capture age relationship	df	AIC<br>Anti-Tc IgA	Anti-Tc IgE	Anti-Tc IgG	Total IgG
Linear	11	3785.757	5024.150	-1262.293	**413.857**
Quadratic	12	3581.726	5015.169	-1268.256	421.198
t=1	12	3784.700	5028.231	-1256.585	415.623
t=2	12	3756.213	5022.865	-1259.245	422.604
t=3	12	3609.695	5016.847	-1266.481	418.428
t=4	12	**3565.230**	**5010.707**	-1268.923	416.371
t=5	12	3582.783	5011.462	-1271.509	414.525
t=6	12	3606.926	5013.595	**-1274.488**	414.664
t=7	12	3633.448	5016.151	-1273.291	414.759
t=8	12	3664.685	5017.673	-1268.788	416.669
t=9	12	3726.003	5023.726	-1260.136	417.943

Table S5. LMM results of models investigating associations between neonatal antibody levels and endogenous antibody levels in 4 month old Soay lambs. Included are the estimated effects (estimate), standard error (SE) and the significance of fixed effects based on a likelihood ratio test (LRT, p-value) where d.f.=1. For the added fixed effects, each of the neonatal antibody measures were added separately to the model. Random effects are quoted for the minimal model excluding added fixed effects.

	**August anti-Tc IgA** n=1773				**August anti-Tc IgE** n=1771				**August anti-Tc IgG** n=1775			
variables	estimate	SE	LRT	P	estimate	SE	LRT	P	estimate	SE	LRT	P
fixed effects												
intercept	-0.250	0.228			-0.266	0.053			-0.434	0.083		
sex (male)	-0.059	0.023	6.644	0.010	0.003	0.006	0.295	0.587	-0.021	0.008	6.130	0.013
twin	0.036	0.032	1.216	0.270	-0.007	0.008	0.889	0.346	-0.016	0.012	2.006	0.157
age (days)	0.009	0.002	19.785	<0.001	0.003	<0.001	44.585	<0.001	0.006	0.001	67.441	<0.001
birth weight	-0.026	0.026	0.975	0.324	0.002	0.006	0.140	0.709	-0.006	0.010	0.398	0.528
added fixed effects												
neonatal anti-Tc IgA	0.034	0.025	1.753	0.186	-0.004	0.006	0.451	0.502	-0.022	0.009	6.262	0.012
neonatal anti-Tc IgE	-0.054	0.018	8.917	0.003	0.001	0.004	0.074	0.785	-0.016	0.006	6.703	0.010
neonatal anti-Tc IgG	-0.164	0.055	8.976	0.003	-0.013	0.013	0.974	0.324	-0.065	0.020	10.933	0.001
neonatal total IgG	-0.191	0.045	18.334	<0.001	-0.014	0.011	1.808	0.179	-0.065	0.016	15.377	<0.001
random effects												
maternal ID	0.044				0.001				0.003			
year	0.011				0.001				0.003			
residual	0.195				0.012				0.029			

Table S6. LMM and GLMM results of models investigating associations between neonatal antibody levels and August weight and August strongyle FEC respectively in Soay lambs. Included are the estimated effects (estimate), standard error (SE) and the significance of fixed effects based on a likelihood ratio test (LRT, p-value) where d.f.=1. For the added fixed effects, each of the neonatal antibody measures were added separately to the model. Random effects are quoted for the minimal model excluding added fixed effects.

	**August weight** n=1770				**August strongyle FEC** n=1528			
variables	estimate	SE	LRT	P	estimate	SE	LRT	P
fixed effects								
intercept	2.817	0.829			6.749	0.389		
sex (male)	1.130	0.074	216.570	<0.001	0.405	0.041	96.164	<0.001
twin	-1.460	0.127	126.490	<0.001	0.108	0.053	4.188	0.041
birth weight	2.451	0.102	503.840	<0.001				
age (days)	0.068	0.007	94.183	<0.001	0.006	0.003	3.042	0.081
maternal age	0.871	0.073	-	-				
maternal age^2	-0.077	0.006	156.760	<0.001				
august weight					-0.085	0.009	87.505	<0.001
august anti-Tc IgA					-0.223	0.040	30.567	<0.001
added fixed effects								
neonatal anti-Tc IgA	0.511	0.086	34.780	<0.001	-0.046	0.042	1.236	0.266
neonatal anti-Tc IgE	0.302	0.064	21.961	<0.001	0.037	0.029	1.614	0.204
neonatal anti-Tc IgG	1.524	0.185	66.485	<0.001	-0.076	0.096	0.626	0.429
neonatal total IgG	1.174	0.146	63.498	<0.001	0.074	0.079	0.872	0.351
random effects								
maternal ID	0.792				0.064			
year	0.732				0.131			
residual	1.940				-			

Table S7. GLMM results of the final minimal model for neonatal and first winter survival including the estimated effects (estimate), standard error (SE) and the significance of fixed effects based on a likelihood ratio test (LRT, p-value) where d.f.=1. For the added fixed effects, each neonatal antibody measure was added separately to the minimal model and significance tested alone. Where interaction terms are significant, estimated effects are stated for the individual terms with the interaction term in the model.

	**Neonatal survival** n=2910				**Winter survival** n=1630			
variables	estimate	SE	LRT	P	estimate	SE	LRT	P
fixed effects								
intercept	4.505	0.390			0.414	0.378		
sex (male)	-0.426	0.213	4.066	0.044	-0.999	0.145	-	-
twin	0.830	0.295	8.414	0.004				
birth weight	1.121	0.150	73.325	<0.001				
maternal age	-0.374	0.106	12.257	<0.001				
august weight					1.057	0.124	-	-
sex (male) * weight					-0.345	0.153	4.995	0.025
added fixed effects								
neonatal anti-Tc IgA	0.443	0.131	13.175	<0.001	0.084	0.071	1.345	0.246
neonatal anti-Tc IgE	0.277	0.138	4.399	0.036	0.068	0.070	0.936	0.333
neonatal anti-Tc IgG	0.649	0.123	29.477	<0.001	0.172	0.073	5.527	0.019
neonatal total IgG	0.475	0.096	22.989	<0.001	0.063	0.073	0.718	0.397

References

1. Sparks AM, Watt K, Sinclair R, Pilkington JG, Pemberton JM, Johnston SE, McNeilly TN, Nussey DH. 2018 Natural Selection on Antihelminth Antibodies in a Wild Mammal Population. *Am. Nat.* **192**, 745–760. (doi:10.1086/700115)

2. Sparks AM, Watt K, Sinclair R, Pilkington JG, Pemberton JM, McNeilly TN, Nussey DH, Johnston SE. 2019 The genetic architecture of helminth-specific immune responses in a wild population of Soay sheep (Ovis aries). *PLoS Genet.* **15**, e1008461. (doi:10.1371/journal.pgen.1008461)

3. Tizard IR. 2012 *Veterinary Immunology*. 9th Ed. St. Louis, Missouri: Elsevier.

4. Hayward AD, Pilkington JG, Wilson K, McNeilly TN, Watt KA. 2019 Reproductive effort influences intra-seasonal variation in parasite-specific antibody responses in wild Soay sheep. *Funct. Ecol.* **33**, 1307–1320. (doi:10.1111/1365-2435.13330)

5. Clutton-Brock TH, Price OF, Albon SD, Jewell PA. 1992 Early Development and Population Fluctuations in Soay Sheep. *J. Anim. Ecol.* **61**, 381–396.

6. Hayward AD, Wilson AJ, Pilkington JG, Clutton-Brock TH, Pemberton JM, Kruuk LEB. 2011 Natural selection on a measure of parasite resistance varies across ages and environmental conditions in a wild mammal. *J. Evol. Biol.* **24**, 1664–1676. (doi:10.1111/j.1420-9101.2011.02300.x)

7. Coltman DW, Pilkington JG, Smith JA, Pemberton JM. 1999 Parasite-mediated selection against inbred Soay sheep in a free-living, island population. *Evolution* **53**, 1259–1267. (doi:10.2307/2640828)

8. Craig BH, Tempest LJ, Pilkington JG, Pemberton JM. 2008 Metazoan-protozoan parasite co-infections and host body weight in St Kilda Soay sheep. *Parasitology* **135**, 433–441. (doi:10.1017/S0031182008004137)

9. Coltman DW, Pilkington J, Kruuk LEB, Wilson K, Pemberton JM. 2001 Positive genetic correlation between parasite resistance and body size in a free-living ungulate population. *Evolution* **55**, 2116–2125. (doi:10.1111/j.0014-3820.2001.tb01326.x)

10. Clutton-Brock TH, Stevenson IR, Marrow P, MacColl AD, Houston AI, McNamara JM. 1996 Population fluctuations, reproductive costs and life-history tactics in female Soay sheep. *J. Anim. Ecol.* **65**, 675–689. (doi:10.2307/5667)
